# Characteristics and short- and long-term direct medical costs among adults with timely and delayed presentation for HIV care in the Netherlands

**DOI:** 10.1371/journal.pone.0280877

**Published:** 2023-02-08

**Authors:** Stephanie Popping, Lisbeth Versteegh, Brooke E. Nichols, David A. M. C. van de Vijver, Ard van Sighem, Peter Reiss, Suzanne Geerlings, Charles A. B. Boucher, Annelies Verbon, on behalf of the ATHENA observational cohort

**Affiliations:** 1 Department of Viroscience, Erasmus Medical Center, Rotterdam, the Netherlands; 2 Department of Medical Microbiology and Infectious Diseases, Erasmus Medical Center, Rotterdam, the Netherlands; 3 Department of Global Health, Boston University, Boston, MA, United States of America; 4 Health Economics and Epidemiology Research Office, Department of Internal Medicine, School of Clinical Medicine, Faculty of Health Sciences, University of the Witwatersrand, Johannesburg, South Africa; 5 Department of Medical Microbiology, Amsterdam University Medical Center, University of Amsterdam, Amsterdam, the Netherlands; 6 Stichting HIV Monitoring, Amsterdam, the Netherlands; 7 Internal Medicine, Division of Infectious Diseases, Center for Infection and Immunity Amsterdam, Amsterdam University Medical Center, Amsterdam, the Netherlands; 8 Department of Global Health, Amsterdam University Medical Centers, University of Amsterdam and Amsterdam Institute for Global Health and Development, Amsterdam, the Netherlands; 9 Department of Internal Medicine and Infectious Diseases, University Medical Center Utrecht, Utrecht University, Utrecht, the Netherlands; University of Catania: Universita degli Studi di Catania, ITALY

## Abstract

**Introduction:**

In Europe, half of people living with HIV (PLWH) present late to care, with associated higher morbidity and mortality. This study aims to assess short- and long-term costs of HIV-care based on time of presentation and identify other factors contributing to higher costs in the first and fifth year after antiretroviral therapy (ART) initiation.

**Material and methods:**

We included ATHENA cohort data which prospectively includes 98% of PLWH in the Netherlands. PLWH who initiated ART in 2013 were included and followed over five years. PLWH were divided in three categories based on CD4 cell-count at time of ART initiation: timely presentation (CD4>350cells/μL), late presentation (CD4 200-350cells/μL or >350cells/μL with AIDS-defining illness) and very late presentation (CD4<200cells/μL). The total HIV-care cost was calculated distinguishing ART medication and non-ART medication costs (hospitalization, outpatient clinic visits, co-medications, and HIV-laboratory tests).

**Results:**

From 1,296 PLWH, 273 (21%) presented late and 179 (14%) very late. Nearly half of those who entered HIV-care in a very late stage were of non-Dutch origin, with 21% originating from sub-Saharan Africa. The mean cost per patient in the first year was €12,902 (SD€11,098), of which about two-thirds due to ART (€8,250 (SD€3,142)). ART costs in the first and fifth year were comparable regardless of time of presentation. During the first year on treatment, non-ART medication costs were substantially higher among those with late presentation (€4,749 (SD€8,009)) and very late presentation (€15,886 (SD€ 21,834)), compared with timely presentation (€2,407(SD€4,511)). Higher non-ART costs were attributable to hospitalization and co-medication. The total non-ART costs incurred across five years on treatment were 56% and 246% higher for late and very late presentation respectively as compared to timely presentation.

**Conclusion:**

Very late presentation is associated with substantial costs, with non-ART costs nearly seven times higher than for those presenting timely. Hospitalization and co-medication costs are likely to continue to drive higher costs for individuals with late presentation into the future. Programs that identify individuals earlier will therefore likely provide significant short- and long-term health cost savings.

## Introduction

Late presentation, or accessing care with a CD4 cell-count <350 cells/μL or with an AIDS-defining illness, has several consequences on an individual clinical, public health, and health systems level [1]. On the individual clinical level, those presenting late in HIV-care often experience higher morbidity and mortality [2, 3]. In addition, a lower CD4-cell count results in a higher risk for opportunistic infections and AIDS-defining malignancies, including Kaposi’s sarcoma, non-Hodgkin lymphoma, and cervical cancers [4]. Moreover, non-AIDS defining malignancies, cardiovascular, renal, and hepatic events appear more frequently in late presenters [4, 5]. From a public health perspective, individuals who present late in HIV-care are mostly unaware of their infection and can continue transmitting HIV to others [6, 7]. Late presentation, therefore, have a large impact on epidemiological control efforts. On a health systems level, those presenting at a later stage in HIV-care are more likely to have prolonged hospitalization, extra laboratory tests, and outpatient visits [8–10]. Even if there is a recovery in health, direct medical costs are estimated to remain higher over time [11, 12].

In 2018, 5.6 per 100,000 people were newly infected with HIV in the European region [13]. Despite several diagnostic improvements and awareness campaigns, approximately half of newly diagnosed people living with HIV (PLWH) presented late in HIV-care and started with antiretroviral therapy (ARTs) at a lower CD4-cell count [14–16].

The comprehensive cost of late presentation has not been assessed with a longer duration of follow-up since the World Health Organization recommended to initiate ART at CD4 cell-count <500μL [17]. We therefore assessed both the short-term (one year) and long-term (five years) costs over five years following ART initiation dependent on time of presentation during the natural course of HIV-infections. Furthermore, we identified the factors contributing to high short-term non-ART costs for direct HIV-care in a resource-rich setting.

## Material and methods

In the Netherlands, HIV-care is provided by designated treatment centres As an integral part of HIV-care, the HIV Monitoring Foundation is responsible for prospectively collecting demographic data and relevant HIV and treatment data, as well as data on viral hepatitis coinfection, from PLWH in the Netherlands and receiving care in one of these 27 treatment centres [18].

This data collection is known as the ATHENA cohort which was initiated in 1998 and captures data from >98% of all patients with a diagnosed HIV-infection who are in care in the Netherlands [19]. Data collection is continuous, and the database of the ATHENA cohort is locked and updated twice a year.

At its inception, the ATHENA cohort was approved by the institutional review boards of all participating centres. Individuals can opt out after being informed by their treating physician of the purpose of data and sample collection. Data are pseudonymized and made available to investigators in a coded form. Coded data may be used for scientific purposes without further consent. For our analysis, only existing data have been used and therefore no additional review or consent was required.

At the end of 2013, 18,000 PLWH in the Netherlands were in care [16]. Men-who-have-sex-with-men (MSM) represented 71% of new diagnoses, making the Dutch HIV epidemic similar to that in other high-income countries [13, 16].

### Study design and population

We included data from PLWH from the Athena cohort who had initiated ART between 1 January 2013 and 31 December 2013 with a follow-up of five years after ART initiation (with the last data included from 31 December 2018). PLWH who enrolled the SHM database more than six months after ART initiation and individuals below the age of 18 years were excluded. PLWH were divided into three groups based on CD4 cell-count at time of ART initiation: timely presentation (CD4 cell count >350cells/μL), late presentation (CD4 cell-count <350 cells/μL or >350cells/μL with an AIDS-defining illness), and very late presentation (CD4 cell-count <200cells/μL). An AIDS-defining illness is defined according to the CDC guidelines.

To describe our patient population and characterize differences between timely, late- and very-late presentation, we conducted univariate analyses using the chi-square, fisher test and unpaired t-test.

### Cost analysis

The total costs of HIV-care were assessed during the first year on ART, beginning at the day of ART initiation. The total long-term costs were assessed from ART initiation and calculated over five years. All health care resources utilized during the respective periods was included. These costs included: ART medication, outpatient clinic visits, laboratory tests (viral load and CD4 cell count measurement), co-medications, and inpatient-days (S1 Table) [20]. The quantity and type of these resources used across the respective follow-up periods was calculated. This was then multiplied by the respective unit costs. The full list of ART prices and co-medication drug prices is reported in the S2 and S3 Tables [20].

Costs are reported in 2018 Euro and presented as cost per patient per year (using means and standard deviations).

### Ethics

At initiation, the cohort was approved by the institutional review board of all participating centers. People entering HIV care receive written material about participation in the ATHENA cohort and are being informed by their treating physician of the purpose of collection of data, after which they can consent verbally or elect to opt-out. Data are pseudonymized before being provided to investigators and may be used for scientific purposes. A designated quality management coordinator safeguards compliance with the European General Data Protection Regulation [19].

## Results

**Fig 1:**
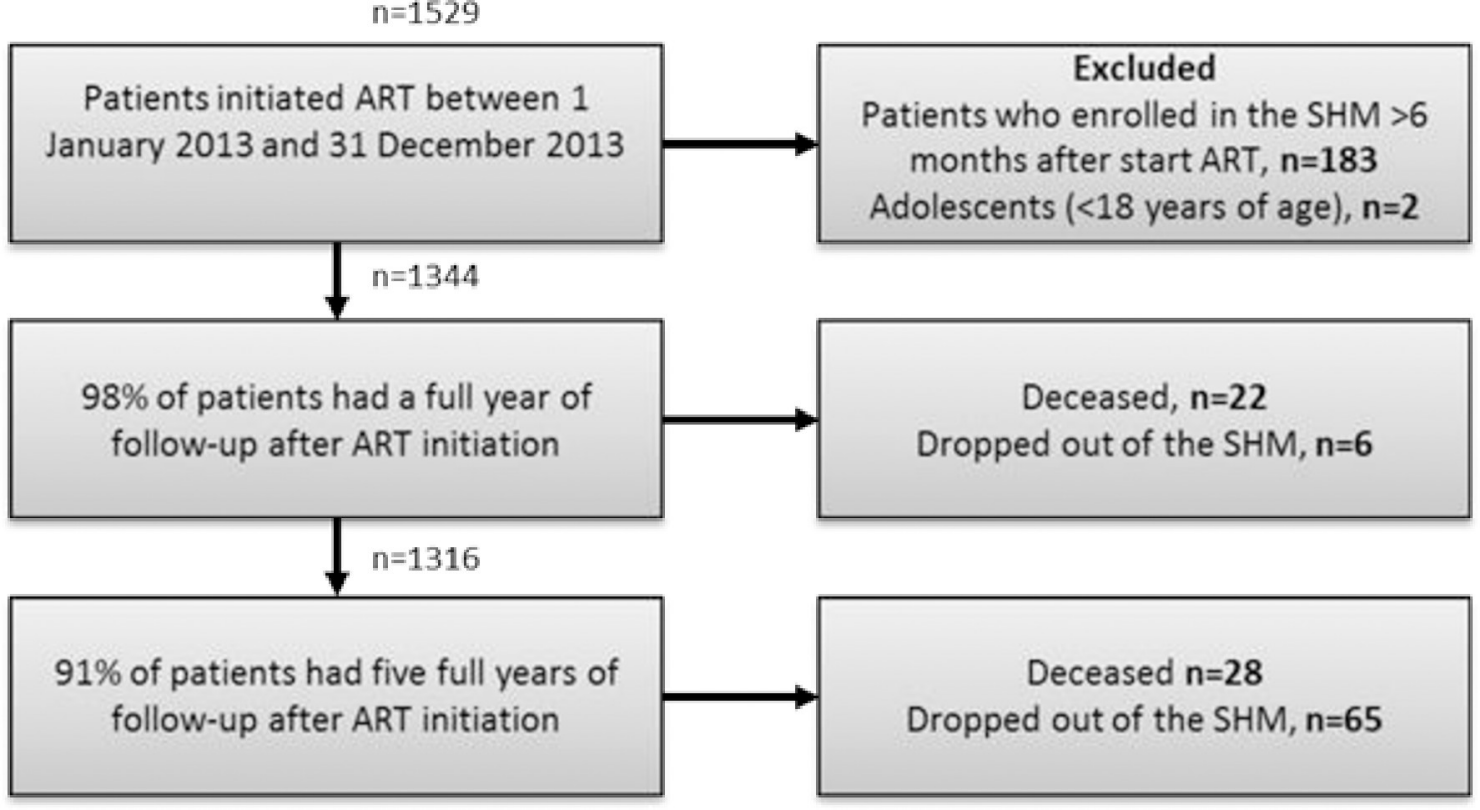
Flow chart of the study. 1223 PLWH remained with complete five years of follow-up data.

In total 1529 PLWH initiated ART between 1 January 2013 and 31 December 2013. After exclusion of PLWH who enrolled the SHM database > 6 months after start of ART and adolescents (<18 years of age), a full year of follow-up was available for 1316 of PLWH (Fig 1). Of PLWH who did not have a full year of follow-up 22 were deceased and 6 dropped-out of the SHM database. During the subsequent four years of follow-up, 28 PLWH died and 65 dropped-out of the SHM, resulting in 1223 of PLWH with five full years of follow-up.

The majority of PLWH included in our analysis were male, comparable to the overall PLWH population in the Netherlands that year [16]. The majority of the PLWH, regardless of time of presentation, were between 40 and 60 years of age. Younger PLWH (<40 years) tended to present timely in care, whereas older PLWH (>60 years) more often presented with a very late stage of HIV-infection (p<0.001).

HIV is mainly transmitted among MSM (Table 1). Individuals who entered HIV-care at a very late stage often acquired HIV through heterosexual contact (p<0.001). PLWH presented most timely when from Dutch origin (70%). Almost half (46%) of those with very late presentation were of non-Dutch origin including Sub-Saharan Africa (21%) and Central- or South America (8%) (p<0.001). PLWH who presented in HIV-care with lower CD4 cell-counts had a shorter period until ART initiation compared with those presented with CD4 cell-counts >350 cells/μL. Viral suppression rates were most successful among individuals with timely presentation. Of the individuals with timely presentation 75% achieved viral suppression within 6 months after ART initiation compared with 64% and 51% with late presentation and very late presentation, respectively (p<0.001). Over half of the individuals with very late presentation switched their ART regimen during the first year of treatment compared to one-third of the individuals presenting timely (p<0.001). Cancer mostly occurred among PLWH with very late presentation in the first year after ART initiation. In contrast, in the four consecutive years cancer was mostly present among those presenting timely (p<0.001). Of the PLWH with very late presentation, more PLWH died (p<0.001) and dropped out of the Athena cohort (p<0.001) during the first year and cumulative over five years.

**Table 1:** Baseline characteristics of n = 1296 PLWH who initiated ART between 1 January 2013 and 31 December 2013.

Number (%)*	Timely presentation	Late presentation	Very late presentation	P-value
	n = 844	n = 273	n = 179	
**Demographic characteristics**				
Gender				
*Male*	754 (89)	240 (88)	149 (83)	0.07
*Female*	90 (11)	33 (12)	30 (17)	
Age				
*<40*	256 (30)	69 (25)	40 (22)	<0.001
*40–60*	498 (59)	159 (58)	96 (54)	** **
*>60*	90 (11)	45 (16)	43 (24)	* *
Transmission mode				
*MSM*	671 (80)	185 (68)	86 (48)	<0.001
*Heterosexual*	147 (17)	64 (23)	67 (37)	* *
Other**	26 (3)	24 (9)	26 (15)	* *
Origin	* *	* *	* *	* *
*NL*	590 (70)	183 (67)	97 (54)	<0.001
*Central/South America*	73 (9)	31 (11)	14 (8)	* *
*Northern Africa*	25 (3)	9 (3)	6 (3)	
*Sub-Saharan Africa*	44 (5)	18 (7)	37 (21)	
*Other^1^*	108 (13)	32 (12)	24 (13)	
**Clinical characteristics**				
Viral load				
*Suppressed ≤6 months*	634 (75)	174 (64)	92 (51)	<0.001
Cancer^2^				
*In first year on ART*	18 (2)	4 (1)	24 (13)	<0.001
*Following four years*	59 (7)	21 (8)	6 (3)	<0.001
Switch				
*In first year on ART*	281 (33)	100 (37)	101 (56)	<0.001
Time from diagnosis to ART				
*Median days [IQR]*	367 [74–1123]	78 [29–832]	23 [13–55]	<0.001
**Patient outcomes**				
Deceased				
*Year 1*	4 (0.5)	3 (1)	14 (8)	<0.001
*Cumulative at year 5*	17 (2)	8 (3)	23 (13)	<0.001
Dropped-out				
*Year 1*	1 (0)	1 (0.4)	4 (2)	<0.001
*Cumulative at year 5*	34 (4)	13 (5)	19 (11)	<0.001

*CD4 cell-count information was unavailable for 48 patients

**Includes: injecting drug user, transfusion (non-hemophilia related), perinatal, homo/hetero unspecified sexual contact, other including medical/dental treatment, needle accident, tattoo, piercing, circumcision, or acupuncture, and unknown.

^1^Includes: Asia, Europe, Aruba, Belarus, Canada, Israel, Japan, Russian Federation, and the USA.

^2^Only non-AIDS defining malignancies

### Resource utilization

During the first year after ART initiation PLWH with very late presentation used the most healthcare resources (Table 2). The strongest differences in the first year after ART initiation was inpatient days: 0.38 (SD 2.3), 1.8 (SD 8.2), 8.3 (SD 23.7) for presenting timely, late, and very late, respectively. Those presenting late and very late also had slightly greater numbers of viral load counts, CD4 cell counts, and outpatient visits as compared to timely presentation. Across all resource categories, with the except of ART-days, PLWH with late and very late presentation utilized more cumulative resources over the five-year time period as compared to those presenting timely.

**Table 2:** Resource utilization during the first and cumulative at five years on ART, based on moment of presentation in HIV-care.

Resource utilization			
*Mean (SD)*	Timely presentation	Late presentation	Very late presentation
	n = 844	n = 273	n = 179
**First year resources used (n = 1,296)**			
ART days	355.6 (45.8)	359.1 (33.1)	337.8 (82.2)
*Viral loads*	3.6 (1.2)	4.0 (1.4)	4.3 (1.7)
*CD4 cell count*	2.7 (1.3)	3.5 (1.6)	4.0 (2.2)
*Outpatient visits*	4.4 (1.9)	5.4 (2.5)	5.9 (3.2)
*Inpatient days*	0.38 (2.3)	1.8 (8.2)	8.3 (23.7)
**Cumulative resources used (n = 1,223)**			
ART days	1821.4 (51.9)	1820.3 (63.0)	1823.2 (20.7)
*Viral loads*	12.9 (3.1)	13.7 (3.6)	14.7 (3.2)
*CD4 cell count*	8.0 (3.1)	9.4 (3.5)	11.0 (3.8)
*Outpatient visits*	14.6 (4.6)	16.2 (5.2)	17.9 (5.8)
*Inpatient days*	1.6 (9.3)	3.3 (9.3)	9.3 (25.5)

### Short-term HIV-care cost increased when presenting late in care

**Fig 2:**
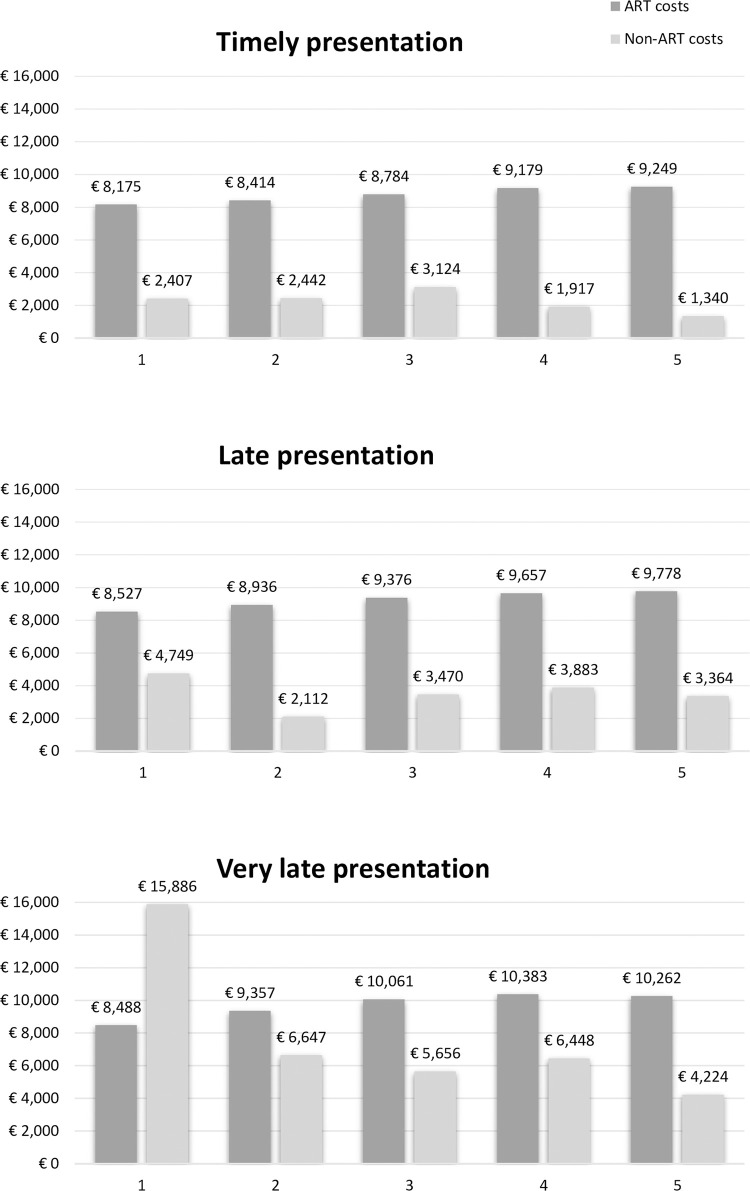
The mean HIV-care cost per individual divided by ART and non-ART related costs according to different stages of presentation for HIV-care. The y-axis represents the cost in € and the x-axis the years after ART initiation.

After the first year of ART initiation the total HIV-care cost per individual is €12,902 (SD€11,098), of which €8,250 (SD€3,142) was for ART medication and €4,652 (SD10,457) for all non-ART medication costs (Fig 2). There is a great variety of the total HIV-care cost based on the time of presentation over the course of HIV-infections mainly driven by non-ART costs (Fig 2). The non-ART costs, including co-medications, hospitalization, viral load, CD4 measurements, and outpatient visits, were an average of €2,407 (SD€4,511) per individual for those presenting timely (Fig 2). For individuals presenting timely in care the non-ART related costs were mostly due to co-medications, €810 (SD€4,084). Non-ART costs increased remarkably to €4,749 (SD€8,009) for individuals presenting late and even more to €15,886 (SD€21,834) very late presentation (Table 3). The total non-ART costs incurred in the first year on treatment were 97% and 560% higher for late and very late presentation respectively as compared to timely.

**Table 3:** Non-ART costs, by resource component and years after ART initiation divided by time of presentation in HIV-care.

	Non-ART cost divided over several cost components per year and time of presentation in HIV-care					
**Timely presentation**						
	**Year 1**	**Year 2**	**Year 3**	**Year 4**	**Year 5**	**Total**
*Co-medication*	€ 810	€ 1,188	€ 2,091	€ 1,064	€ 614	€ 5,767
*Viral loads*	€ 438	€ 304	€ 297	€ 269	€ 247	€ 1,555
*Visit*	€ 574	€ 355	€ 346	€ 319	€ 273	€ 1,868
*Inpatient days*	€ 261	€ 407	€ 216	€ 128	€ 87	€ 1,099
*Outpatient days*	€ 324	€ 189	€ 175	€ 137	€ 117	€ 942
***Total***	€ 2,407	€ 2,442	€ 3,124	€ 1,917	€ 1,340	€ 11,231
**Late presentation**						
*Co-medication*	€ 1,895	€ 793	€ 2,206	€ 2,737	€ 2,335	€ 9,966
*Viral loads*	€ 487	€ 335	€ 313	€ 273	€ 240	€ 1,648
*Visit*	€ 697	€ 416	€ 369	€ 320	€ 293	€ 2,094
*Inpatient days*	€ 1,256	€ 331	€ 383	€ 407	€ 358	€ 2,735
*Outpatient days*	€ 414	€ 237	€ 198	€ 146	€ 121	€ 1,116
***Total***	€ 4,749	€ 2,112	€ 3,470	€ 3,883	€ 3,346	€ 17,560
***Difference in cost compared to timely presenters***	97%	-14%	11%	103%	150%	56%
**Very late presentation**						
*Co-medication*	€ 8,491	€ 4,704	€ 3,980	€ 4,951	€ 3,067	€ 25,193
*Viral loads*	€ 524	€ 350	€ 320	€ 288	€ 233	€ 1,715
*Visit*	€ 760	€ 447	€ 369	€ 344	€ 284	€ 2,205
*Inpatient days*	€ 5,644	€ 873	€ 776	€ 698	€ 515	€ 8,506
*Outpatient days*	€ 467	€ 273	€ 211	€ 167	€ 126	€ 1,242
***Total***	€ 15,886	€ 6,647	€ 5,656	€ 6,448	€ 4,224	€ 38,862
***Difference in cost compared to timely presentation***	560%	172%	81%	236%	215%	246%

Similar as to timely presentation non-ART costs were mostly attributed to the use of co-medications. However, co-medication costs were twice the amount (€1,895 (SD€4,195)) for presentation late and ten times the amount (€8,491 (SD€10,729)) for presenting very late. Additionally, hospitalization costs are more common among individuals with late presentation (€1,256 (SD€5,565)) and very late presentation (€5,644 (SD€16,074)), while negligible for those who presented timely (€261 (SD€1,564)) (Table 3).

### Long-term HIV-care cost remained high for individuals presenting at a later stage in care

Over the years, the total annual HIV-care cost per individuals remained similar for PLWH with a timely presentation (range €10,289 - €11,768) (Fig 2). Although the total HIV-care cost decreased for presenting late- and very late after the first year on ART, the total HIV-care cost was 24% higher for presenting late and 19% higher for very late as compared to timely presentation at year five on ART (€12,721 and €12,221 respectively, compared with €10,289). Non-ART costs continued to drive the variation between the different patient groups, while ART costs remained stable over time (Fig 2). The long-term non-ART costs for timely presentation were an average of €1,270(SD€3,536) per individual, mostly due to co-medications (Table 3). The total non-ART costs incurred across five years on treatment were 56% and 246% higher for late and very late presentation respectively as compared to timely presentation.

## Discussion

Our results showed that apart from higher morbidity, mortality, and prolonged possibility of transmission, late presentation- and more specifically very late presentation- resulted in considerably higher short-term HIV-care costs. The higher cost per patient that initiated timeously compared to those with a late presentation persisted through to the end of our follow-up period at five years post ART initiation. Although the cost of ART medication comprises the greatest proportion of the total annual HIV-care costs, non-ART costs drive the increasing cost of late presentation. The short-term non-ART costs were nearly twice as high for late presentation and nearly seven-times higher for very late presentation as compared with those who presented timely in HIV-care. Over a five-year period, the total HIV-care cost for late presentation were 56% higher for late presentation and 246% higher for very late presentation as compared to timely presentation.

Cost components driving the higher short-term non-ART cost among PLWH presenting late in care not surprisingly mainly included hospitalization and co-medication costs. Given the likelihood of such individuals suffering from conditions requiring frequent and prolonged hospitalization and the use of multiple co-medications. Unfortunately, after five years this increase in costs is still present mainly in the form of increased co-medication costs. This suggests that even though the clinical condition of PLWH presenting later in care improved, over time their health state remains worse than those presenting timely. Similar findings were found by Krentz et al. who found higher continuing medical costs for PLWH presenting later in care related to non-ART drugs [11].

We identified several patient characteristics associated with late presentation such as heterosexual transmission, elderly, and origin in line with other studies [14, 15, 21]. Late presentation is often driven by postponed HIV testing due to low risk perception, lack of HIV awareness, or stigma, and can largely vary over patient groups [14, 15, 22]. Especially among elderly acquiring HIV is often unconsidered due to the enduring misconception that mostly young people acquire HIV resulting in insufficient testing in this population group [22, 23]. In developed countries, however, almost half of PLWH are aged 50 or older and account for 17% of new HIV-infections. Moreover, they are more likely than younger adults to already have AIDS at the time of diagnosis. Therefore, increased awareness among both older individuals themselves and their health care providers may result in earlier case finding.

Another strategy which may help in earlier identification of HIV disease, would be through HIV indicator condition related testing [24, 25]. HIV indicator conditions are conditions associated with HIV infection through similar risk factors or appear with an impaired immune system [26]. HIV indicator condition testing is considered effective and often present among those who presented late in HIV-care [26–28]. Moreover, it normalises HIV-testing and does not requires a risk assessment.

Migrants, from low- and middle-income countries, are a subgroup which are disproportionately affected by HIV in high-income countries and frequently present late for care High levels of HIV-related stigma, unawareness, and limited access to care result in insufficient engagement with HIV prevention and treatment services. Therefore migrants are placed at risk of poor HIV related clinical outcomes [29]. In order to reach this unaware population more aggressive testing could be performed by for example HIV indicator condition testing or implement the distribution of HIV self-tests [30–32].

Free and easy to access self-tests could also lower the barrier for stigma among several risk groups [30]. In addition, HIV awareness on early testing in disproportionately affected subgroups should be increased among general practitioners and other healthcare workers. This can be generated by organizing programs such as the Amsterdam HIV Testing Week, where large scale HIV-testing was implemented resulting in a positive response among targeted risk groups, including non-Western migrants [33]. Large scale HIV-testing programs stress the importance of structural free of charge low-threshold HIV-testing. Furthermore, partner notification is another suitable and cost-effective strategy that may help to identify new infections in an earlier stage [34].

Apart from the individual clinical benefits of timely presentation into HIV-care, our findings stress the importance of earlier identification of HIV-infection from a cost perspective in both the short and long run. Moreover, investing in earlier identification will also results in a decrease of transmission. Importantly, given that an individual presenting late incurs €8,463 and very late incurs €26,404 of additional costs over the first five years on treatment compared to those presenting timeously, these financial resources could be redirected to novel testing and HIV screening programmes. If a new HIV-infection could be identified for that cost, then those testing or screening programmes would quickly become cost-saving, particularly since the cost savings that we calculate here do not include the cost of onward transmission.

A major strength of our study is that the population studied can be considered to be highly representative of the PLWH and in care in the Netherlands, as more than 98 percent of those in care consent to their data being collected as part of the ATHENA cohort [19]. As 91% of our study population had a five- year follow-up this data provided a unique and solid opportunity to calculate the long-term economic burden of HIV-disease and the impact of presenting late for care. The distribution of mode of transmission in the Netherlands is representative of other high-income countries, making our results generalizable across the region.

Our study was the first study analyzing the long-term follow-up after ART treatment is recommended for patients with CD4 cell-counts <500 μL [17]. The guidelines of September 2015, however, changed and recommended immediately initiate ART after diagnosis of HIV. As half of our PLWH presented late in care and could therefore not benefit from the effect of early ART initiation. The effect of immediate ART initiation would, however, lower the cost of timely presenters and subsequently increasing the cost of patients presenting late and very late in care. As our study calculated cost from the time of ART initiation our HIV care cost may even be an underestimation. Individuals with a timely presentation may have several hospital visits before starting on ARTs and those with late- or very late presentation could already be hospitalized for opportunistic infections prior to ART initiation.

One of the limitations of this study is that we did not have access to all costs of HIV-care as appointments or procedures performed by a general practitioner are not included. This could result in a slight under-estimation of costs for timely presentation, since they possibly use more care provided by general practitioners. Care provided by a general practitioner is, however, less expensive than hospital care. Moreover, our resource utilization showed that PLWH with timely presentation only have slightly less outpatient visits. Therefore, it would be unlikely that visits to the general practitioner office would make a substantial difference. Secondly, we did not include cost to the patient. If considering costs from the patient perspective, those presenting very late, due to the greater number of inpatient days and outpatient visits incurred, are likely to have incurred more personal costs than those presenting on time. Including costs from the patient perspective would only have further strengthened our conclusions. Lastly, we excluded PLWH who died during the study period as we calculated a full year and consecutive full four years of HIV health care utilization. The number of PLWH who died that presented very late in care was much higher compared to individuals with timely and late presentation even after five years. Although these PLWH utilized less health care costs, due to short hospital admission stay and often not receiving ARTs, they have the worst health care outcome, death. This further strengthens the need for programs focused on early identification of new HIV infections.

## Conclusion

In conclusion, late presentation can result in substantial additional non-ART-medication costs over one- and five-year time periods. Several patient characteristics as country of origin, transmission route, and age are predictors of presenting late in care highlighting populations where aggressive testing strategies should be implemented, and awareness should be increased, to maximize both individual benefit and reduce costs. Investment in these strategies could improve clinical outcomes, prevent onward HIV transmission, and avoid these long-term excess patient costs.

## Supporting information

S1 Table Cost of resources. Costs are presented per unit in €.
(DOCX)

S2 Table Cost of co-medication. The cost of co-medications per day in €. Costs are calculated based on the average of the lowest and highest price of the available medication for a 70kg adult and 2018 list price.
(DOCX)

S3 Table Cost of ART. The cost of ART per day in €. Costs are calculated based on the average of the lowest and highest price of the available medication and 2018 list price.
(DOCX)
