# Supplementary material for: Characteristics and short- and long-term direct medical costs among adults with timely and delayed presentation for HIV care in the Netherlands
Source: PLoS One. 2023 Feb 8;18(2):e0280877. doi: 10.1371/journal.pone.0280877 (PMC9907815; doi:10.1371/journal.pone.0280877)
Supplement: S1 Table — Costs are presented per unit in €. (DOCX) [file pone.0280877.s001.docx]

**Supporting Information**

**S1 Table. Cost of resources**

|  |  |
| --- | --- |
| **Resources** | |
| Price per unit (€) |  |
| *Hospitalization day* | 679 |
| *Outpatient care visit* | 129 |
| *CD4 cell count* | 118 |
| *HIV viral load measurement* | 121.5 |

**Table 1**: cost of resources. Costs are presented per unit in €
