# Supplementary material for: Characteristics and short- and long-term direct medical costs among adults with timely and delayed presentation for HIV care in the Netherlands
Source: PLoS One. 2023 Feb 8;18(2):e0280877. doi: 10.1371/journal.pone.0280877 (PMC9907815; doi:10.1371/journal.pone.0280877)
Supplement: S2 Table — The cost of co-medications per day in €. Costs are calculated based on the average of the lowest and highest price of the available medication for a 70kg adult and 2018 list price. (DOCX) [file pone.0280877.s002.docx]

**Supporting Information**

**S2 Table. Cost of co-medication**

| **ATC-code** | **Price (€)** |
| --- | --- |
| A02AA02 | 0.16 |
| A02AA04 | 0.16 |
| A02AB02 | 1.23 |
| A02AD | 0.94 |
| A02AD01 | 1.38 |
| A02BA02 | 0.79 |
| A02BA03 | 0.76 |
| A02BC01 | 0.36 |
| A02BC02 | 2.69 |
| A02BC04 | 0.25 |
| A02BC05 | 2.90 |
| A02BD04 | 10.75 |
| A03AA04 | 0.31 |
| A03BA01 | 5.52 |
| A07AA02 | 1.50 |
| A07AA08 | 11.76 |
| A07AC01 | 10.93 |
| A07DA03 | 1.43 |
| A10AB01 | 1.12 |
| A10AB05 | 3.00 |
| A10AC04 | 5.22 |
| A10AD05 | 1.16 |
| A10AE05 | 11.33 |
| A10BA02 | 0.16 |
| A10BB01 | 0.15 |
| A10BB03 | 0.18 |
| A10BB09 | 0.14 |
| A10BB12 | 0.08 |
| A10BG03 | 0.49 |
| A10BH01 | 3.12 |
| A10BX02 | 0.61 |
| A11CC | 0.02 |
| A11CC05 | 0.02 |
| A11HA02 | 1.02 |
| A12AA04 | 0.51 |
| A12AX | 0.51 |
| A14AB01 | 21.06 |
| B01AA04 | 0.08 |
| B01AA07 | 0.10 |
| B01AB01 | 25.80 |
| B01AB04 | 7.30 |
| B01AB05 | 5.80 |
| B01AB06 | 3.12 |
| B01AB10 | 10.52 |
| B01AC04 | 0.79 |
| B01AC06 | 0.58 |
| B01AC07 | 0.50 |
| B01AC08 | 0.07 |
| B01AC22 | 2.81 |
| B01AC24 | 2.49 |
| B01AD04 | 134.50 |
| B01AE07 | 3.68 |
| B01AX05 | 4.54 |
| B01AX06 | 2.81 |
| B02AA02 | 4.76 |
| B02BX04 | 832.04 |
| B03AA02 | 0.47 |
| B03AA07 | 0.09 |
| B03BB01 | 0.03 |
| C01AA05 | 0.71 |
| C01BA02 | 0.78 |
| C01BC04 | 3.86 |
| C01BD01 | 1.67 |
| C01CE02 | 17.97 |
| C01DA02 | 0.24 |
| C01DA08 | 0.36 |
| C01DA14 | 0.24 |
| C01EB17 | 1.88 |
| C02AB01 | 0.81 |
| C02AC01 | 0.70 |
| C02CA04 | 0.17 |
| C02DC01 | 1.76 |
| C03AA03 | 0.07 |
| C03BA04 | 0.10 |
| C03CA01 | 0.49 |
| C03CA02 | 0.57 |
| C03DA01 | 0.16 |
| C05AA01 | 0.09 |
| C05AA12 | 0.05 |
| C05AE03 | 0.04 |
| C05AX03 | 0.39 |
| C05AX04 | 0.08 |
| C05BA01 | 0.05 |
| C07AA05 | 0.38 |
| C07AA07 | 14.68 |
| C07AB02 | 0.92 |
| C07AB03 | 0.10 |
| C07AB07 | 0.42 |
| C07AB09 | 73.07 |
| C07AB12 | 0.26 |
| C07AG01 | 4.95 |
| C08CA01 | 0.06 |
| C08CA04 | 17.19 |
| C08CA05 | 0.34 |
| C08CA12 | 0.71 |
| C08CA13 | 0.13 |
| C08DA01 | 1.35 |
| C08DB01 | 0.27 |
| C09AA01 | 0.10 |
| C09AA02 | 0.05 |
| C09AA03 | 0.52 |
| C09AA04 | 0.27 |
| C09AA05 | 0.16 |
| C09AA06 | 0.15 |
| C09AA09 | 0.11 |
| C09BA01 | 0.11 |
| C09BA02 | 0.48 |
| C09BA03 | 0.11 |
| C09BA04 | 0.28 |
| C09CA01 | 0.13 |
| C09CA03 | 0.12 |
| C09CA04 | 0.32 |
| C09CA06 | 0.21 |
| C09CA07 | 0.40 |
| C09DA01 | 0.20 |
| C09DA03 | 1.73 |
| C09DA04 | 0.19 |
| C09DA07 | 0.25 |
| C09DB01 | 0.81 |
| C09DX01 | 1.05 |
| C09DX04 | 5.08 |
| C09XA02 | 0.76 |
| C10AA01 | 0.19 |
| C10AA03 | 0.14 |
| C10AA04 | 0.22 |
| C10AA05 | 0.70 |
| C10AA07 | 0.35 |
| C10AB04 | 0.60 |
| C10AC01 | 1.38 |
| C10AX09 | 0.42 |
| C10BA02 | 1.52 |
| D01BA02 | 0.11 |
| D02AB | 0.04 |
| D04AB01 | 0.12 |
| D05AX52 | 0.34 |
| D06BB10 | 0.14 |
| D07AB02 | 0.05 |
| D07XA01 | 0.09 |
| D07XC01 | 0.06 |
| D10BA01 | 0.69 |
| G01AF02 | 1.53 |
| G02BA03 | 0.09 |
| G02BB01 | 0.48 |
| G03A | 0.10 |
| G03AA07 | 0.12 |
| G03AA09 | 0.09 |
| G03AA13 | 3.14 |
| G03AC03 | 1.45 |
| G03AC06 | 14.83 |
| G03AC08 | 0.11 |
| G03BA03 | 1.45 |
| G03BE30 | 0.00 |
| G03CA03 | 6.26 |
| G03FB08 | 0.23 |
| G03GB02 | 0.10 |
| G03HA01 | 2.56 |
| G03HB01 | 0.08 |
| G04BD04 | 1.38 |
| G04BD07 | 0.70 |
| G04BD08 | 0.70 |
| G04BE03 | 7.98 |
| G04BE08 | 8.15 |
| G04BE09 | 3.51 |
| G04CA01 | 0.31 |
| G04CA02 | 0.22 |
| G04CA53 | 1.19 |
| G04CB02 | 0.38 |
| H02AB02 | 4.87 |
| H02AB04 | 18.57 |
| H02AB06 | 6.36 |
| H02AB07 | 0.11 |
| H02AB08 | 6.49 |
| H02BA09 | 0.00 |
| H03AA01 | 1.71 |
| J01 | 0.00 |
| J01AA02 | 2.20 |
| J01AA08 | 0.82 |
| J01BA01 | 0.11 |
| J01CA04 | 1.79 |
| J01CE01 | 9.64 |
| J01CE08 | 15.95 |
| J01CF05 | 4.73 |
| J01CR02 | 4.32 |
| J01CR05 | 11.32 |
| J01DC02 | 3.41 |
| J01DD01 | 4.97 |
| J01DD04 | 12.12 |
| J01DH02 | 17.52 |
| J01EA01 | 0.53 |
| J01EC02 | 1.39 |
| J01EE01 | 17.33 |
| J01FA01 | 8.74 |
| J01FA09 | 0.79 |
| J01FA10 | 1.41 |
| J01FF01 | 5.65 |
| J01GB01 | 22.97 |
| J01GB03 | 11.04 |
| J01MA01 | 1.34 |
| J01MA02 | 11.61 |
| J01MA12 | 13.44 |
| J01MA14 | 19.88 |
| J01XA01 | 11.76 |
| J01XC01 | 4.52 |
| J01XE01 | 0.55 |
| J02AA01 | 81.27 |
| J02AB02 | 10.93 |
| J02AC01 | 18.36 |
| J02AC02 | 121.62 |
| J02AC03 | 58.32 |
| J02AX01 | 49.65 |
| J02AX04 | 372.38 |
| J04AB02 | 4.81 |
| J04AB04 | 3.55 |
| J04AC01 | 0.33 |
| J04AD01 | 0.33 |
| J04AK01 | 1.28 |
| J04AK02 | 1.15 |
| J04AM02 | 1.24 |
| J04BA01 | 0.17 |
| J04BA02 | 0.17 |
| J05AB01 | 10.91 |
| J05AB04 | 11.17 |
| J05AB06 | 35.42 |
| J05AB11 | 10.81 |
| J05AB14 | 29.57 |
| J05AD01 | 166.70 |
| J05AE11 | 287.20 |
| J05AE12 | 107.70 |
| J05AE14 | 382.61 |
| J05AP57 | 467.61 |
| J05AX14 | 433.11 |
| J05AX15 | 379.55 |
| J05AX16 | 457.86 |
| J05AX65 | 481.73 |
| J05AX67 | 457.86 |
| J05AX68 | 272.50 |
| J05AX69 | 321.61 |
| J06AX09 | 83.94 |
| J06BA02 | 1258.95 |
| L01 | 0.00 |
| L01AA01 | 30.64 |
| L01AA02 | 2.14 |
| L01AA06 | 86.39 |
| L01AD02 | 7.77 |
| L01AX04 | 55.05 |
| L01BA0 | 582.72 |
| L01BA01 | 116.36 |
| L01BA04 | 875.51 |
| L01BC01 | 80.78 |
| L01BC02 | 18.81 |
| L01BC05 | 130.91 |
| L01BC06 | 115.46 |
| L01CA01 | 38.15 |
| L01CA02 | 16.64 |
| L01CA03 | 16.64 |
| L01CB01 | 38.15 |
| L01CB02 | 38.15 |
| L01CD01 | 736.80 |
| L01DB01 | 540.81 |
| L01DB03 | 193.14 |
| L01XA01 | 25.89 |
| L01XA02 | 115.90 |
| L01XC02 | 1117.15 |
| L01XE01 | 14.32 |
| L01XX05 | 10.47 |
| L02BA01 | 0.32 |
| L03AA02 | 71.31 |
| L03AA13 | 890.40 |
| L03AB1 | 44.71 |
| L03AB10 | 143.29 |
| L03AB11 | 143.29 |
| L04AA05 | 35.65 |
| L04AX01 | 0.66 |
| M01AE02 | 0.46 |
| M04AC01 | 0.51 |
| M05BA04 | 0.46 |
| M05BA06 | 38.18 |
| N01AH02 | 2.28 |
| N01AH03 | 10.91 |
| N01AH06 | 13.05 |
| N01BB52 | 1.37 |
| N01BX04 | 302.58 |
| N02AA01 | 2.17 |
| N02AA05 | 3.74 |
| N02AB02 | 1.02 |
| N02AB03 | 19.92 |
| N02AC03 | 1.85 |
| N02AD01 | 8.88 |
| N02AE01 | 4.68 |
| N02AX02 | 0.77 |
| N02AX52 | 0.77 |
| N02CA52 | 1.85 |
| N03AB02 | 9.31 |
| N03AE01 | 0.77 |
| N03AF01 | 0.85 |
| N03AG01 | 1.21 |
| N03AX09 | 15.54 |
| N03AX11 | 3.00 |
| N03AX12 | 0.92 |
| N03AX14 | 7.26 |
| N03AX16 | 2.06 |
| N05AD01 | 7.73 |
| N05AG02 | 0.37 |
| N05AH02 | 1.41 |
| N05AH03 | 0.84 |
| N05AH04 | 2.45 |
| N05AX08 | 1.01 |
| N05AX12 | 2.28 |
| N05BA01 | 1.03 |
| N05BA05 | 0.24 |
| N05BA06 | 0.43 |
| N05BA12 | 0.23 |
| N05CD08 | 2.56 |
| N05CF01 | 0.17 |
| N06AA04 | 0.31 |
| N06AA09 | 0.32 |
| N06AA10 | 0.51 |
| N06AB03 | 0.20 |
| N06AB04 | 0.40 |
| N06AB05 | 0.61 |
| N06AB06 | 0.62 |
| N06AB08 | 0.32 |
| N06AB10 | 0.31 |
| N06AX05 | 0.97 |
| N06AX11 | 0.26 |
| N06AX12 | 1.93 |
| N06AX16 | 0.56 |
| N06AX21 | 0.65 |
| N06BA02 | 2.97 |
| N06BA04 | 1.68 |
| N06DX02 | 0.28 |
| N07BB01 | 0.21 |
| N07BC02 | 0.47 |
| P01AB01 | 4.99 |
| P01AX06 | 29.51 |
| P01BA02 | 0.48 |
| P01BA03 | 0.48 |
| P01BB51 | 12.56 |
| P01BD01 | 1.25 |
| P01BE03 | 16.27 |
| P01CX01 | 35.14 |
| P02BA01 | 52.35 |
| P02CA01 | 0.69 |
| P02CA03 | 3.76 |
| P02CF01 | 18.37 |
| R01AD08 | 1.03 |
| R01AD58 | 0.32 |
| R03AC13 | 1.05 |
| R03AK06 | 0.80 |
| R03AK07 | 1.37 |
| R03BA01 | 1.06 |
| R03BA02 | 0.54 |
| R03BA08 | 0.53 |
| R03DA04 | 0.14 |
| R06AD02 | 0.49 |
| R06AX13 | 0.37 |
| S01CA01 | 0.21 |
| S01EA05 | 0.18 |
| S01EB01 | 0.84 |
| S01EC01 | 0.54 |
| S01EC03 | 0.30 |
| S01ED01 | 3.88 |
| S01ED51 | 0.41 |
| S01EE01 | 2.09 |
| S01EE03 | 5.71 |
| V03AF03 | 83.40 |
| V03AF07 | 231.26 |

**Table 2:** The cost of co-medications per day in €. Costs are calculated based on the average of the lowest and highest price of the available medication for a 70kg adult and 2018 list price^1^.

**References**

1. Medication cost (Medicatie Kosten). 2022. 2022, at <www.medicijnkosten.nl>.)
