# Supplementary material for: Characteristics and short- and long-term direct medical costs among adults with timely and delayed presentation for HIV care in the Netherlands
Source: PLoS One. 2023 Feb 8;18(2):e0280877. doi: 10.1371/journal.pone.0280877 (PMC9907815; doi:10.1371/journal.pone.0280877)
Supplement: S3 Table — The cost of ART per day in €. Costs are calculated based on the average of the lowest and highest price of the available medication and 2018 list price. (DOCX) [file pone.0280877.s003.docx]

**Supporting Information**

**S3 Table. Cost of ART**

| **ATC-code** | **Price (€)** |
| --- | --- |
| J05AE01-SQH | 10.70 |
| J05AE01-SQS | 10.70 |
| J05AE03 | 10.80 |
| J05AE04 | 26.00 |
| J05AE07 | 13.41 |
| J05AE08 | 12.96 |
| J05AE10 | 16.81 |
| J05AF01 | 8.08 |
| J05AF04 | 7.18 |
| J05AF05 | 5.00 |
| J05AF06 | 7.89 |
| J05AF07 | 6.17 |
| J05AF08 | 13.80 |
| J05AF09 | 6.29 |
| J05AF10 | 6.42 |
| J05AF11 | 14.48 |
| J05AF13 | 11.71 |
| J05AG01 | 5.29 |
| J05AG03 | 7.58 |
| J05AG04 | 14.51 |
| J05AG05 | 9.09 |
| J05AR01 | 5.29 |
| J05AR02 | 6.06 |
| J05AR03 | 5.24 |
| J05AR04 | 20.47 |
| J05AR05 | 10.57 |
| J05AR06 | 15.50 |
| J05AR07 | 17.47 |
| J05AR08 | 23.83 |
| J05AR09 | 35.22 |
| J05AR10 | 15.69 |
| J05AR11 | 18.75 |
| J05AR13 | 32.2 |
| J05AR14 | 14.83 |
| J05AR15 | 14.05 |
| J05AR17 | 17.69 |
| J05AR18 | 32.45 |
| J05AR19 | 24.93 |
| J05AR20 | 31.25 |
| J05AR21 | 29.03 |
| J05AR22 | 30.98 |
| J05AR24 | 17.68 |
| J05AX08 | 18.02 |
| J05AX09 | 40.37 |
| J05AX12 | 20.89 |
| L01XX05 | 57.97 |
| V03AX03 | 1.09 |
| J05AX09 | 40.37 |
| J05AX12 | 20.89 |
| L01XX05 | 5.79 |
| V03AX03 | 1.09 |

**Table 3:** The cost of ART per day in €. Costs are calculated based on the average of the lowest and highest price of the available medication and 2018 list price^1^.

**References**

1. Medication cost (Medicatie Kosten). 2022. 2022, at <www.medicijnkosten.nl>.)
